# Academic health science networks' experiences with rapid implementation practice during the COVID-19 pandemic in England

**DOI:** 10.3389/frhs.2022.943527

**Published:** 2022-08-04

**Authors:** Alexandra Ziemann, Andrew Sibley, Harry Scarbrough, Sam Tuvey, Sarah Robens

**Affiliations:** ^1^Centre for Healthcare Innovation Research, City, University of London, London, United Kingdom; ^2^Wessex Academic Health Science Network, Southampton, United Kingdom; ^3^Bayes Business School, City, University of London, London, United Kingdom; ^4^South West Academic Health Science Network, Exeter, United Kingdom; ^5^Re!nstitute, Stamford, CT, United States

**Keywords:** rapid implementation, COVID-19, healthcare, social care, innovation, implementation strategies, context, mechanisms

## Abstract

The COVID-19 pandemic offered a “natural laboratory” to learn about rapid implementation of health and social care innovations in an altered implementation context. Our aim was to explore implementation practice of Academic Health Science Networks (AHSN) in the English National Health System during the first wave of the COVID-19 pandemic through a rapid implementation lens. We organized three 90-min, online, semi-structured focus groups with 26 operational and senior managerial staff from 14 AHSNs in June-July 2020. Participants were recruited purposefully and on a voluntary basis. Participants presented a case study about their approaches to implementing innovations between March-June 2020 and discussed their experiences and lessons learned. The focus groups were audio-recorded and transcribed verbatim. Transcripts and other documents were analyzed using qualitative thematic analysis following a combination of grounded theory and framework analysis approach. AHSNs increased the pace of their implementation work to support the response to the COVID-19 pandemic. The disruptive event changed the implementation context which enabled rapid implementation through an urgency for change, the need to adhere to social distancing rules, new enabling governance structures, and stakeholders' reduced risk averseness toward change. AHSNs achieved rapid implementation through: (1) An agile and adaptive implementation approach; (2) Accelerating existing innovations and building on existing relationships/networks; (3) Remote stakeholder engagement; and (4) Ensuring quality, safety, rigor and sustainability, and generating new evidence through rapid evaluations. AHSNs aimed at sustaining implementation pace and efficiency after the acute phase of the pandemic mainly through remote stakeholder engagement and flexibility of implementation strategies.

## Introduction

Rapid approaches seem particularly pertinent in implementation science to reach the field's underlying goal of closing the know-do gap (1). Smith et al. (2) have recently set out the first theoretical conceptualization of rapid implementation, defined as achieving “speed and efficiency, by redefining rigor, and adapting both methods [and] design” (p. 9). The approach holds the promise of a paradigm shift to close the knowledge-to-practice gap more efficiently and rapidly (2). It might allow those involved in implementing changes to “fail fast” and quickly adapt their implementation strategy to the dynamic real-world contextual changes characterizing the complex health and social care systems in which implementation takes place (1, 3, 4). Smith et al.'s (2) conceptualization is focusing on accelerating the research process, however, our understanding how to apply it in implementation practice is only just starting to emerge.

Rapid approaches applied in implementation science are covering mainly rapid data collection and analysis methods. For example, Davis and colleagues (2021) reflected on the application of rapid ethnography for contextual assessments in implementation studies (5), and Last et al. (3) described a pilot study of rapid prototyping for refining implementation strategies. Other examples for rapid methods relevant to implementation science are rapid randomized controlled trials, rapid qualitative analyses, rapid-cycle evaluations, or rapid review methods (6–10).

Some studies have started to emerge which are reporting on sudden contextual changes resulting in the need for rapid implementation in practice with the prime example being the COVID-19 pandemic which functioned as a catalyst for the implementation of innovations (11). By innovation, we are referring to any change to existing practice that is new to a location ranging from products to practice or process changes (12). Reports about rapid implementation often referred to an accelerated implementation of innovations which have not been taken up in the past or not as fast, and driven by the need of adhering to social distancing rules, e.g., digital, virtual or remote interventions (13–17).

The COVID-19 pandemic offers a “natural laboratory” and a unique window of opportunity to learn about practice experiences with rapid implementation. This would contribute empirical knowledge to further develop our understanding of the concept and improve implementation practice after the acute phase of the pandemic (18, 19). We were interested to learn from the activity and experiences of intermediary organizations, Academic Health Science Networks (AHSNs), which are officially mandated with facilitating the implementation of innovations in the English National Health System (NHS). The aim of this study was to explore AHSN implementation practice experiences during the first wave of the COVID-19 pandemic through a rapid implementation lens.

## Materials and methods

### Design

The study applied a qualitative case study design. We are reporting findings according to the Consolidated Criteria for Reporting Qualitative Research (COREQ) guidelines and the COREQ checklist can be found in Supplement 1. The study is part of a wider study “Review of Spread and Adoption Approaches across the AHSN Network” which explored the general implementation and spread activity at AHSNs before and during COVID-19 (20, 21). Ethical approval was obtained from City, University of London, The Business School Research Ethics Committee (ETH1920-1032).

### Setting

The study focused on analyzing implementation activity by staff working at AHSNs in England. AHSNs were set up by NHS England in 2013 and relicensed in April 2018 to operate as the key innovation arm of NHS England (22). They are regional intermediary organizations designed to support their local health and social care system to implement innovations at pace and scale, improve population health and generate economic growth. There are 15 AHSNs in England with on average around 40–50 part- and full-time staff members, each covering a distinct geography. All AHSNs have the same commissioners (NHS England and NHS Improvement and the United Kingdom Government's Office for Life Sciences) resulting in all AHSNs having to fulfill the same national policy framework and mandate and following a broadly similar pattern of innovation and implementation activity (23). Within this framework, they have been largely free to develop their own specific implementation approaches and strategies.

### Participant selection and recruitment

Focus group participants were purposively selected based on their expertise and experience in being involved in an operational or senior management role in the implementation of innovations during the period of March-June 2020. We limited recruitment to a maximum of two participants per AHSN (preferably one operational and one senior management participant) to keep the focus groups manageable and reduce burden on AHSNs and staff members to participate in this study while being under particular work pressures during the pandemic. At each of the 15 AHSNs, the research team had (senior) management contacts who nominated participants for focus groups and provided contact details of participants to the research team. Recruitment of participants was conducted by AZ *via* email. Participation was voluntary and participants could withdraw from the study before data analysis was completed. Participants were provided with information about the study and were asked to provide written consent before the focus groups.

### Data collection

Focus groups were conducted by all authors *via* Zoom in a remote working/home office context (this study was conducted under a COVID-19 pandemic work-from-home-if-possible advice by the UK Government applying to research team and participants). Two technical support staff members from South West AHSN were present in addition. The focus groups were conducted applying a semi-structured discussion guide asking for participant's activity and experiences implementing health innovations during the same three-month period between March and June 2020. The discussion guide was driven by the following questions: What has been the experience of implementation by AHSNs (activities taken and changes in the environments they are conducting spread work within), and what impacts have been seen during the COVID-19 pandemic (Supplement 2). During the focus groups, all participants presented a case study about their approaches to implementing different types of innovations between March-June 2020, and discussed their experiences and lessons learned in the group. The guide was pilot-tested by AS with staff at Wessex AHSN who were not participants. Focus groups were audio-recorded and transcribed verbatim.

### Data analysis

Data from the transcripts, presentation slides and additional documents shared by participants on their implementation activity were analyzed applying qualitative thematic analysis. We applied two rounds of coding with the first following a grounded theory approach to inductively identify themes around the changed implementation context, AHSN implementation activity and experiences of AHSN staff. This led to the clear insight that AHSNs were engaging in rapid implementation and we coded the data in a second round applying a framework analysis approach using the rapid implementation conceptualization by Smith et al. (2) in combination with a realist-type approach deriving themes around context, practice (realist-type mechanism) and outcomes (24). We identified themes around contextual characteristics enabling rapid implementation (realist-type context: changes to implementation context as consequence of the COVID-19 pandemic), of speed and efficiency (realist-type outcome: characteristics of rapid implementation as implementation outcome), and of redefined rigor and adapted methods (realist-type mechanism: implementation practice aimed at achieving rapid implementation). The coding tree for the second round of coding can be found in Supplement 3. Coding was conducted by AS, AZ, HS, and SR using NVivo 1.0 (25). To ensure trustworthiness and validity of our analysis, we used a de-briefing technique in the research team with one member conducting the initial analysis around one topic area, e.g., implementation context, and the other members reviewing the analysis and providing feedback which was discussed in the group until consensus was reached.

### Data synthesis

Themes were synthesized by AS, AZ, HS, and SR in narrative and graphic form. Synthesis was guided by a realist approach (24), as we aimed at deriving a realist-type context-mechanism-outcome statement to provide an initial mid-range theory on the practice application of rapid implementation. The synthesis was checked by two different external groups, one being the wider project's advisory group consisting of senior AHSN and NHS England representatives, and the other being the senior executive leaders of all AHSNs who were overseeing the AHSNs' spread work during COVID-19. We discussed and incorporated the received feedback.

### Research team and reflexivity

The research team consisted of two researchers based at City, University of London (AZ, PhD, female, senior research fellow, and HS, PhD, male, full professor) and three researchers based at two AHSNs, Wessex AHSN (AS, PhD, male, evaluation programme manager) and South West AHSN (SR, PhD, female, evaluation lead and spread fellow, and ST, MSc/PhD, male, evaluation researcher). AHSN research team members added a unique inside-perspective and understanding concerning general implementation activity in an AHSN environment that was complemented with the external view brought in by the university-based research team members. AHSN research team members have a professional work relationship with some of the participants but were not involved in recruiting participants. Research team members (i.e., name, affiliation, occupation, role in study) and the goals of the study were introduced to participants during recruitment and again at the beginning of the focus groups. The research team have a health services and social science background with AS and AZ applying an implementation science lens and HS applying an organization studies lens to innovation research. All research team members have training and experience in applying qualitative methods and conducting focus groups.

## Results

Twenty-six staff from 14 out of the 15 AHSNs participated in three 90-min focus groups between 29 June and 7 July 2020. Four participants dropped out due to competing work priorities. Twelve senior management and 14 operational staff members participated with between seven and eleven participants per focus group. Participants reported cases of implementing a range of different types of innovations related to service or process innovations, e.g., redesigned outpatient pathways, medical products, e.g., new diagnostic tests, or digital solutions, e.g., virtual clinics.

**Figure 1:**
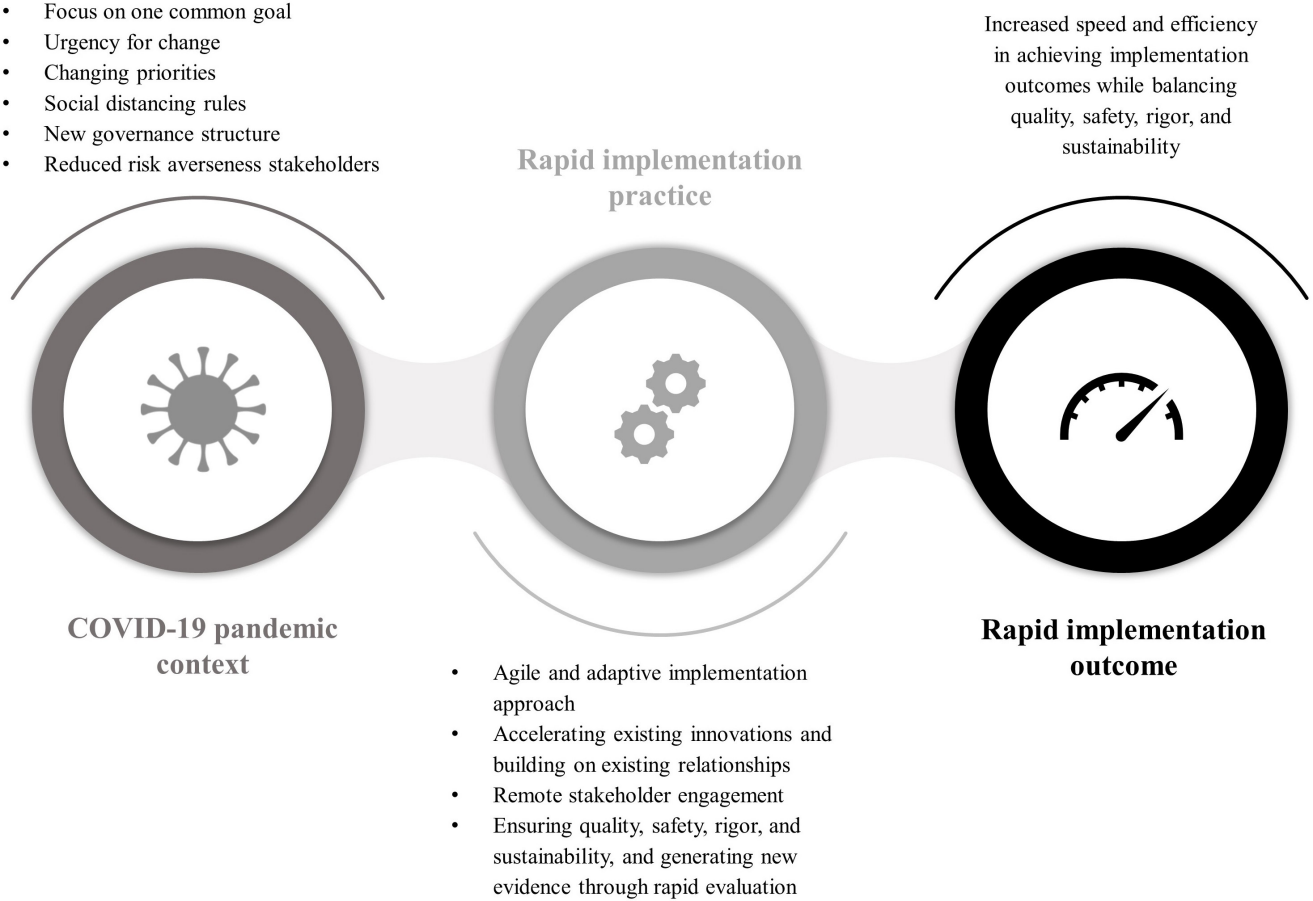
Realist-type statement presenting an initial mid-range theory for rapid implementation practice applied by AHSNs during the COVID-19 pandemic. The COVID-19 pandemic posed a disruptive event influencing the implementation context in such a way that it increased the need for and was enabling rapid implementation. The changed context was characterized by the focus on one common goal, i.e., managing the pandemic, an urgency for change, a change in priorities in terms of focusing on certain health and care settings or types of innovations, the implementation of social distancing rules, new governance structures, and a reduced risk averseness of stakeholders. Rapid implementation as outcome was characterized by an increased speed and efficiency in achieving implementation outcomes while balancing quality, safety, rigor, and sustainability. AHSNs changed their implementation practice in response to the altered context and with the aim to achieve rapid implementation. Rapid implementation practice was characterized by a move to more of an agile and adaptive implementation approach, the acceleration of existing innovations and building on existing stakeholder relationships, remote stakeholder engagement, and ensuring safety, quality, rigor and sustainability, and generating new evidence through rapid testing and evaluation.

Figure 1 summarizes the findings of the thematic analysis in the form of a realist-type statement representing an initial mid-range theory for the practice application of rapid implementation by AHSNs during the COVID-19 pandemic. In the following, we first report how the implementation context for AHSNs changed after the start of the pandemic and how this enabled or resulted in rapid implementation practice. Afterwards, we report on changes in implementation practice applied by AHSNs in response to the changed implementation context to achieve rapid implementation.

### COVID-19 created an enabling context for rapid implementation

AHSNs saw a sudden change in focus with stakeholders rallying around the common goal of responding to the pandemic. There was also an urgency for change with the need to find solutions to manage the pandemic quickly. While AHSNs were faced with supporting the whole health and care system, there was a change in emphasis on certain healthcare settings over others, changing the focus of the AHSNs' work. For example, at the beginning of the pandemic there was an increased focus on care homes.

New rules, mainly around social distancing, came into force with very short notice. This changed the way how healthcare organizations worked but also how the AHSNs worked themselves, which required rapid solutions enabling remote care and remote working. This enabled the implementation of innovations which might not have been taken up widely beforehand, e.g., telehealth applications.

AHSNs saw a change in the mindset in those involved in implementing innovations in terms of accepting risk. It was more important to have a solution quickly, even if that meant accepting solutions that might not have been proven for a particular setting or use case.

“*I think the pandemic, if nothing else, has created that sort of urgency within the system and a bit more risk-taking and less risk-aversion, which I think as long as we*'*re rigorous and diligent with governance, is great.” (Participant Focus Group 2)*

AHSNs also recognized a change in governance structures in the NHS with new structures being put in place to enable quick decision making at national and regional policy levels. This accelerated decision-making process was enabled also by the reduction in risk averseness mentioned above. Certain governance processes and structures which posed barriers to implementation before the pandemic were abandoned as they were standing in the way to take quick necessary action. AHSNs felt more empowered and engaged by the changed governance structures who recognized AHSNs, competence and invited them into the decision-making processes or permitted them to take decisions.

“*If you get a signal from [the national policy level] it's okay to crack on, that empowers us to do it, whereas so often before then it's always been you can't do it.” (Participant Focus Group 2)*

While participants reported how the changed context enabled implementation work, they also reported on mounting pressures and difficulties managing competing priorities for them and in the health system, particularly at the early stages of the pandemic.

“*The progress has been a lot slower than we anticipated […] because of what was going on in the system, and care homes in particular […], they had loads of staff off sick, they had residents who were dying from COVID on a frequent basis. Although this would have supported [the implementation process] they were firefighting, so there wasn't the resource or the capacity to take on something new.” (Participant Focus Group 1)*

### AHSNs' rapid implementation practice

AHSNs increased the pace and efficiency of their implementation activities to support NHS organizations in their response to COVID-19. AHSNs have achieved rapid implementation through: (1) An agile and adaptive implementation approach; (2) Accelerating existing innovations and building on existing relationships and networks; (3) Remote stakeholder engagement; and (4) Ensuring quality, safety, rigor and sustainability, and generating new evidence through rapid evaluations.

#### Agile and adaptive implementation approach

Given the suddenly changing context and needs of the NHS, AHSNs applied a more agile approach to meeting the changing local needs quickly.

“*We literally just hit the black book and thought ‘who can we call'? Who can we literally pick up the phone to and say ‘clearly you have different needs right now, what are they? How can we help?” (Participant Focus Group 1)*

AHSNs saw a particular need to support care homes which were under heightened pressure at the beginning of the pandemic. Care homes have traditionally not been a focus of AHSN work and they had to quickly adjust to serve this new setting. The agility was also recognized in the ways in which AHSN workforce were working, with staff sometimes being deployed to work directly within healthcare provider organizations or their roles being extended, e.g., taking on more operational work. AHSNs felt the need to work in a way that met the need for rapid action, for example, using rapid testing approaches, and many saw a change in their own attitude of trying something out.

#### Accelerating existing innovations and building on existing relationships and networks

AHSNs relied on existing solutions and their existing networks and relationships to be able to quickly identify, adapt, and implement innovations; e.g., the new focus on care homes and the need for remote care saw AHSNs adapt an existing intervention which had been implemented in hospitals and private home settings in the past to be now extended to care homes.

Existing relationships have been key to ensuring rapid implementation during the pandemic, with a particular emphasis on using relationships to rapidly identify need and offer support. Existing relationships with innovators and suppliers allowed AHSNs to quickly gather a range of possible solutions to meet the need of stakeholders. It also meant that once innovations were identified for implementation, AHSNs could engage with early adopters for rapid feedback to help spread this innovation quickly to other adopters.

#### Remote stakeholder engagement

The necessity of working remotely proved to largely increase the efficiency of AHSNs' implementation activities. Stakeholder engagement, training, and information dissemination was now done online which saved time and was at times more inclusive in terms of reaching a larger audience in a more flexible manner. However, some staff reported that remote engagement wasn't always working for them in terms of building new relationships and offering training.

“*I think [COVID-19] has given permission, […] to just plant meeting requests in people's diaries at really short notice, then use the technology that we're all using now to quickly […] build a relationship with somebody.” (Participant Focus Group 1)*

“*Some of what we were trying to do was around delivering support and training and that kind of thing. Not being able to do that on a face-to-face basis I think is challenging.” (Participant Focus Group 1)*

#### Ensuring quality, safety, rigor and sustainability, and generating new evidence through rapid evaluation

While AHSNs increased their pace in implementing innovations and facilitated the implementation of new solutions they wanted to make sure to build in evaluation to ensure quality and safety, for example, when an innovation was implemented in a new care setting. This also proved to be another window of opportunity to generate new evidence on those new solutions to inform local implementation and sustainability after the acute phase of the pandemic.

“*The evaluation and benefits capture, so commissioners have reassurance that this is not a short-term fix. […] We need to*

*make sure that whatever we've got is sustainable and we don't undo some of the good work that we've been able to do during this crisis period, because it has been an opportunity really to mobilize specifically digital innovations.” (Participant Focus Group 1)*

Some participants reported that the need for rapid implementation had the negative effect of reducing patient and public involvement in testing and evaluating innovations.

### Sustaining rapid implementation practice

Participants discussed if and which rapid implementation activities they would like to maintain after the acute phase of the pandemic. Most AHSN staff welcomed the increase in pace and efficiency of implementation efforts and would like to maintain this, if however, at a more feasible rate as many participants experienced large work pressures from rapid implementation during the pandemic. While acknowledging that many of the enabling contextual changes would not stay in place or would be out of their control, participants aimed at sustaining the increased remote stakeholder engagement and the agility and adaptiveness of implementation strategies.

Participants also reported that they would like to keep the stronger links with other AHSNs and with stakeholders in their region which were enabled by the new governance structures and need for urgent action around a common goal.

## Discussion

We analyzed AHSNs' implementation practice during the first wave of the COVID-19 pandemic in England through a rapid implementation lens. We found that a combination of remote and agile ways of working, adapting existing innovations and building on existing relationships and a changed enabling context allowed for increased speed and efficiency of implementation. While increasing rapid implementation, AHSNs aimed at ensuring quality, safety, rigor, and implementation sustainability by incorporating rapid testing and evaluation.

To our knowledge our study has for the first time contributed insights into how the rapid implementation conceptualization by Smith et al. (2) could be applied in practice. Our findings generally confirm the components of the concept in terms of speed achieved by redefining rigor and adapting methods. Rapid implementation was characterized by an increase in pace and efficiency. It was enabled through the adaptation of implementation approaches, and a change in rigor, mainly characterized by a less restrictive governance context and stakeholder's reduced risk averseness. As also highlighted by Smith et al. (2), our study showed that it was important to maintain a certain level of rigor in terms of ensuring quality and safety of rapidly implemented interventions.

We are providing an initial insight into rapid implementation practice and how implementation activity might be adapted to achieve rapid implementation. It became very clear that agile ways of working and adapting existing interventions for a new purpose played an important role. The latter was mirrored by other rapid implementation cases reported during the pandemic (13, 16, 26). In addition, we found that the increased application of remote stakeholder engagement helped increase implementation efficiency and pace.

Smith et al.'s (2) rapid implementation conceptualization explicitly refers to evidence-based interventions. In contrast, AHSNs were deploying interventions with a limited evidence-base to meet the need for rapid solutions in practice. There is a risk that rapidly implemented non-evidence-based interventions might be harmful or ineffective (11). AHSNs mitigated this risk through the parallel application of rapid testing and evaluation approaches offering the opportunity to ensure quality and safety. Interestingly, these evaluations also contributed to the build-up of the evidence base for new use cases.

Our findings confirm those of other studies in terms of the enabling effect of COVID-19 for rapid implementation (11, 13, 15), and particularly also the implementation of digital and remote technology in healthcare (14, 17). Many of the enabling contextual factors are only in place temporarily, posing the question if and how rapid implementation practice should or will be maintained after this crisis period. We found that rapid implementation did increase work pressure on AHSN staff while they also felt that they would like to keep implementing at pace and more efficiently mainly through engaging stakeholders remotely. Future research should investigate if and how rapid implementation has been operationalized in a non-crisis context. This could entail studying the trade-off's regarding capacity and costs of employing rapid implementation approaches and prioritizing certain innovations at the cost of others.

### Limitations

Our study has two main limitations. The data was only referring to a short period of time at the beginning of the pandemic which was characterized for participants by a highly disruptive change in their ways of working to adjust to the new pandemic context. These unique circumstances while having made our study possible also resulted in our findings being hardly representative of usual practice later in the pandemic or in non-crisis times. Our findings can only be understood as initial insights and validity during non-crisis times has to be confirmed by future research. Further, our study is reflecting the experiences of one group of stakeholders involved in rapid implementation. These are views of intermediaries though, who have a broad overview on implementation activity involving different stakeholders, different types of innovations, different settings, and represent mostly all regions in England. Nevertheless, future studies should involve reflections of other stakeholder groups, in particular service providers and users.

## Conclusions

AHSN staff achieved rapid implementation during the COVID-19 pandemic through agile and adaptive implementation approaches, an increase in remote stakeholder engagement, and a balance of rapid testing and evaluation approaches. These insights from rapid implementation practice have contributed to our understanding of the rapid implementation concept and inform if and how to operationalize rapid implementation in non-crisis times.

## Data availability statement

The raw data supporting the conclusions of this article will be made available by the authors, without undue reservation.

## Ethics statement

The studies involving human participants were reviewed and approved by City, University of London, the Business School Research Ethics Committee (ETH1920-1032). The participants provided their written informed consent to participate in this study.

## Author contributions

AS, AZ, HS, and SR conducted the data analysis and synthesized the data. AZ and SR wrote the first draft of the manuscript. All authors conceived the study design, participated in data collection, revised the first manuscript draft, read, and approved the final manuscript.

## Funding

The reported research was funded by the AHSN Network and the NHS England Innovation, Research and Life Sciences team. The funding bodies had no influence on the design of the study, the collection, analysis, and interpretation of data, and the writing of the manuscript.

## Conflict of interest

The authors declare that the research was conducted in the absence of any commercial or financial relationships that could be construed as a potential conflict of interest.

## Publisher's note

All claims expressed in this article are solely those of the authors and do not necessarily represent those of their affiliated organizations, or those of the publisher, the editors and the reviewers. Any product that may be evaluated in this article, or claim that may be made by its manufacturer, is not guaranteed or endorsed by the publisher.

## Supplementary material

Supplement 1 COREQ checklist.

Supplement 2 Focus group discussion guide.

Supplement 3 Coding tree.

## Abbreviations

AHSN: Academic Health Science Network
COREQ: Criteria for Reporting Qualitative Research
COVID-19: Coronavirus Disease 2019
NHS: National Health System.
