# Supplementary material for: Academic health science networks' experiences with rapid implementation practice during the COVID-19 pandemic in England
Source: Front Health Serv. 2022 Aug 4;2:943527. doi: 10.3389/frhs.2022.943527 (PMC10012785; doi:10.3389/frhs.2022.943527)
Supplement: Supplement 2 — Focus group discussion guide. [file Data_Sheet_2.PDF]

Ziemann et al. - Rapid Implementation

Supplement 2

Focus group discussion guide

**10 minutes**	**Introduction to study and focus group, round of introductions of participants, questions, consent process, Zoom focus group etiquette**	**Plenary**<br>Focus group moderator, technical support
**40 minutes**	**Part 1: AHSN COVID-19 spread/implementation examples and experiences**<br><br>Presenter questions to be covered in presentation:<br>1) Could each AHSN representative talk us through your practical experiences with one example of a new or existing innovation you have spread/implemented during the COVID-19 pandemic, with a focus on:<br>a. How did you go about spreading/implementing this innovation?<br>b. What spread/implementation and adoption outcome did you achieve and how does that relate to the chosen spread/implementation and adoption approach?<br>c. What key barriers and enablers to spreading/implementing the innovation did you encounter?<br>d. What have you learned about spreading/implementing this innovation during the COVID-19 pandemic?<br><br>Group discussion question (after each presentation):<br>e. Has anyone else spread/implemented similar innovations and did you have similar or different spread/implementation and adoption experiences?	**2 break-out groups** (half of the participants each, 8 min per example)<br><br>**5 min** 'presentation' (any format, e.g., 1-2 slides or free speech)<br><br>**3 min** group discussion per example after each presentation and at any time in Zoom chat
**5 minutes**	**Summary feedback from the break-out groups**	**Plenary**<br>Rapporteurs Part 1
**30 minutes**	**Part 2: General focus group discussion comparing experiences during COVID-19 to period before and after COVID-19**<br><br>Discussion questions:<br>2) What differences have you seen in your spread/implementation work during COVID-19 compared to the period of around two years prior?<br>3) What major lesson or what change in your spread/implementation work during COVID-19 would you like to keep for your future spread/implementation work after COVID-19?	**Plenary**<br><br><br>**20 min**<br><br><br>**10 min**
**5 minutes**	**Wrap-up**<br>- Summary of key points from Part 2<br>- Overview on next steps/timelines for study outcomes, especially expected feedback to AHSNs	**Plenary**<br>Focus group chair, Rapporteur Part 2
