# Supplementary material for: Academic health science networks' experiences with rapid implementation practice during the COVID-19 pandemic in England
Source: Front Health Serv. 2022 Aug 4;2:943527. doi: 10.3389/frhs.2022.943527 (PMC10012785; doi:10.3389/frhs.2022.943527)
Supplement: Supplement 3 — Coding tree. [file Data_Sheet_3.PDF]

Ziemann et al. Rapid Implementation

Supplement 3

Coding tree

Category	Codes
Context	Competing demands
	Urgency
	Window of opportunity
	Timelines not aligned
	Confusion, chaos, complexity, difficulties, conflicting priorities
	Change in needs, priorities
	Funding
	Governance and support structures, levers
	Reduction of barriers
	Fit to needs/demands
	Risk averseness reduction
	Permission to experiment
	Action-oriented, just-do-it attitude
	Mindset change
	Common goal
	Reputation AHSN
	Willingness to collaborate
	Acceptance of lower evidence base
	More comfortable using digital innovations/ways of working
	Do the right thing attitude
	Agile decision-making structures
	Volunteer involvement
	Must do attitude
	Redeployment
Rapid Implementation Practice	Accelerating existing innovations
	Adaptation
	Enhancing innovations
	AHSN collaboration increase
	Re-assigning AHSN resources
	Tapping into new governance structures
	Engaging all stakeholders early on
	Remote working
	Use existing networks
	Use existing skills, strategies, information and approaches
	Agile working
	Take over more decision-making/lead at AHSN
	Remote stakeholder engagement
	Use of more Quality Improvement methodology
	Engage right stakeholders
	Piloting
	Local focus
	New innovations
	Rapid decision-making
	New stakeholder engagement
	Easier collaboration
	Safety and quality assurance
	Less rigorous application of structured approach

	Generate evidence
	Evaluation
	Rapid methods
	Ensure sustainability
	Shared learning
	Patient and Public Involvement
Rapid implementation outcome	Speed
	Efficiency
	Uptake
	Penetration
Lessons	Sustainability after COVID
	Advantages, what works
	Disadvantages, what doesn't work
